# Psychometric assessment of the Moroccan version of the car, relax, alone, forget, friends, trouble (CRAFFT) scale among adolescent and young adults with a substance use disorder

**DOI:** 10.1186/s13722-025-00557-y

**Published:** 2025-03-21

**Authors:** Hicham El Malki, Abdelfettah El-Ammari, Salma Ghofrane Moutawakkil, Samir Elgnaoui, Fatima El Houari, Karima El Rhazi, Btissame Zarrouq

**Affiliations:** 1 Laboratory of Epidemiology and Research in Health Sciences, Faculty of Medicine, Pharmacy, and Dental Medicine, Sidi Mohamed Ben Abdellah University, Fez, Morocco; 2 Scientific Innovation in Sustainability, Environment, Education, and Health in the Era of Artificial Intelligence, Teachers Training College (Higher Normal School), Sidi Mohamed Ben Abdellah University, Fez, Morocco; 3 Addictology Center, Fez, Morocco; 4 Psychology Department, Private University, Fez, Morocco

**Keywords:** Alcohol, Drug, CRAFFT, Addictology, Psychometrics, Validity

## Abstract

**Background:**

The Car, Relax, Alone, Forget, Friends, Trouble (CRAFFT) scale is a widely used screening tool for early identification of alcohol and other drug use, and assessing the risk of substance use disorders in adolescents and young adults. Despite its broad use, translation into several languages, and validation in various settings, no study has yet confirmed the psychometric properties of a Moroccan version. The present research aims to adapt and validate the Moroccan Arabic dialect version of the CRAFFT scale among adolescents and young adults with alcohol and drug use disorder.

**Methods:**

A total of 302 adolescents and young adults (mean age = 18.36 ± 2.36), including 161 males and 41 females, were recruited from a substance use treatment center in Fez City. Confirmatory Factor Analysis (CFA) was used to assess the factorial structure and model fit, while internal consistency was evaluated using the Kuder-Richardson Formula 20 (KR-20). Convergent validity was examined using gold standard measures, including the International Neuropsychiatric Interview (MINI) and the Hooked-on Nicotine Checklist (HONC). All statistical analyses were performed using JASP software (version 0.17).

**Results:**

CFA revealed a one-factor structure with a good overall fit (χ²/df = 1.91, Root Mean Square Error of Approximation (RMSEA) = 0.06, Standardized Root Mean Square Residual (SRMR) = 0.03, Comparative Fit Index (CFI) = 0.98, Normed Fit Index (NFI) = 0.97. The model had strong reliability with a KR-20 coefficient of 0.80. Convergent validity was confirmed by a high and significant correlation with the MINI gold standard (*r* = 0.82, *p* < 0.001), while a low correlation with the HONC gold standard (*r* = 0.20, *p* < 0.001) confirmed the scale’s convergent validity. A cutoff score of 4 or higher on the CRAFFT was identified as optimal for balancing sensitivity (78.35%) and specificity (91.67%), achieving a Youden index of 0.70.

**Conclusion:**

The psychometric properties of the Moroccan version of the CRAFFT confirm that it is a valid tool for screening the early detection of alcohol and drug use and for assessing the risk of substance use disorders in adolescents and young adults.

**Supplementary Information:**

The online version contains supplementary material available at 10.1186/s13722-025-00557-y.

## Background

Adolescence, a pivotal stage in development, is frequently typified by the initiation and exploration of behaviors carrying potential health risks, such as substance use experimentation, with these challenges often persisting into young adulthood [1, 2]. Worldwide, this concern represents a contemporary health issue that continues to gain momentum due to the significant increase in consumption among this population [3, 4].

According to current data from 2021, 296 million people worldwide—roughly 1 in 17 people—had used psychoactive substances (PAS) in the preceding year, a 23% increase over 2010. Among individuals with substance use disorders, young people are significantly more vulnerable to drug use than adults, with approximately 5.3% (around 13.5 million individuals) aged between 15 and 16 years [5]. This striking trend indicates notable disparities, with the highest rates observed in the America, Australia, and Africa [6]. Alcohol, cannabis, and tobacco emerge as the predominant and commonly substances used during adolescence, particularly in low- and middle-income countries (LMICs) worldwide, according to findings from the Global School-Based Student Health Survey (GSHS) [7].

In Morocco, despite limited reliable data on PAS use [8], several studies indicate that drug use is relatively common among adolescents and young adults. In the Center-North region, 4.3% of secondary school students reported using alcohol, 1.7% used inhalants, and 1.0% used non-prescribed psychotropic substances [9]. A 2017 study in the central region found lifetime use rates of 17.4% for alcohol, 16.1% for cannabis, and 5.1% for non-prescribed psychotropics among students from various faculties [10]. In the Oriental region, a survey of students at Mohammed I University in Oujda showed that 15.9% had used alcohol, 24.1% had used tobacco, and 13.4% had used cannabis [11]. Among students aged ≤ 20 years, 20.3% reported having used a psychoactive substance at least once in their lifetime, indicating that approximately one in five young individuals in this age group had engaged in substance use. Furthermore, the 2017 Mediterranean School Project on Alcohol and Other Drugs (MedSPAD III) study, which covered all regions, found that 8.0% of individuals aged 15 to 17 had consumed alcohol at least once in their lifetime, while 6.0% reported using alcohol in the past 30 days [12].

Alcohol and drug use among adolescents and young adults significantly contributes to risky behaviors, such as dangerous driving, and is a primary factor in injury, violence, and mortality [13–15]. Prolonged use of these substances is connected to serious health issues, including cancer, cardiovascular and liver diseases [16], and neurological disorders, as well as psychological conditions such as antisocial personality disorder, depression, and anxiety [17–20].

Although the magnitude of problems associated with alcohol and drug use among adolescents and young adults is significant, and despite the development of several tools for the early detection of alcoholism or problematic use in the last 30 to 40 years [21], limited research has been conducted in Morocco to adapt prevention or treatment services specifically based on scientific screening methods [22]. The CRAFFT scale, developed by Knight [23], is one of the most extensively used brief screening tools for detecting alcohol and drug use and related issues among adolescents and young adults. It has been validated across diverse contexts and has demonstrated robust psychometric properties, including high reliability and construct validity [24–28], affirming its efficacy and suitability for informing prevention and treatment strategies, thereby reinforcing its utility in clinical practice [29].

Since its inception, the CRAFFT screening tool has undergone several revisions to enhance its structure, clarity, and relevance in assessing adolescent substance use [25]. CRAFFT 2.0 revised its wording to align with contemporary substance use patterns and is accessible in 18 languages [30]. CRAFFT 2.1 expanded its scope to address emerging concerns such as vaping and prescription drug misuse, while CRAFFT 2.1 + N further incorporated nicotine consumption, including vaping and e-cigarette use. Both CRAFFT 2.1 and CRAFFT 2.1 + N have been translated into 34 languages [30]. In the Moroccan context, where the prevalence of e-cigarette use remains low, the inclusion of nicotine-specific items in CRAFFT 2.1 + N may not be justified [31]. Additionally, the increased number of items in CRAFFT 2.1 + N could compromise the brevity and efficiency required for a screening tool tailored to adolescents and young adults.

Thus, the current study aimed to verify the psychometric characteristics of the CRAFFT, specifically the self-administered version 2.1, among Moroccan adolescent and young adult with alcohol and drugs use disorder.

## Methods

### Simple study/study design

A cross-sectional study involving Moroccan adolescents young adults receiving treatment at a substance use treatment center in the city of Fez was conducted. The study period extended from February 2021 to June 2022.

### Samples/participants

A sample of 302 individuals was selected following the recommended participant-to-variable ratio to ensure the reliability of factor analysis results [32]. Participant recruitment adhered to the 20:1 ratio, aligning with established Confirmatory Factor Analysis (CFA) guidelines and validated by multiple references [33–35].

All study participants were individuals seeking outpatient treatment for use disorder. The primary requirements for their involvement were that they be adolescents and young adult, aged 12 to 21, who had reported using alcohol and other drugs within the previous 12 months. In addition to the recruits’ consent to participate, parental consent has been obtained for participants who are under the age of 18.

### Measures

#### CRAFFT

The CRAFFT scale, whose acronym reflects the essential elements of the tool (Car; Relax; Alone; Forget; Friends; Trouble) is a brief six-question screening instrument designed for use in clinical settings, developed by the Center for Adolescent Substance Abuse Research (CeASAR) [23]. It is designed especially for the early identification of children, adolescents and young adults (12 to 21 years old), who used alcohol or drugs in the last year and might be vulnerable of developing an alcohol or drug use disorders [36–38]. Other established research supports the use of the CRAFFT screening tool with individuals up to the age of 26 [27].

The self-administered CRAFFT 2.1 employs a binary response format, with participants assigning 1 point for ‘Yes’ and 0 points for ‘No [23, 37, 39]. Each respondent who reported a 12-month history of alcohol or other substance use is assigned a score ranging from zero to six. A score of two and above indicated a problematic pattern of usage (use or dependence) [23, 37].

#### Mini-International neuropsychiatric interview (M.I.N.I.)

The Moroccan Colloquial Arabic adaptation of the M.I.N.I. alcohol dependence module was utilized to assess the convergent validity of the CRAFFT screening tool [40]. This DSM-IV-based instrument comprises 12 dichotomous (Yes/No) items, with a cutoff of three or more affirmative responses indicating current alcohol dependence. Psychometric evaluation in Morocco demonstrated high reliability and validity (Cronbach’s α = 0.89, sensitivity = 0.82, specificity = 1.00, PPV = 1.00, NPV = 0.98) [40].

#### Hooked on nicotine checklist (HONC)

The Moroccan adaptation of the HONC (under submission) was employed to establish divergent validity [41]. This 10-item dichotomous (Yes/No) questionnaire assesses loss of autonomy over tobacco use in adolescents and young adults. Scores classify individuals into full autonomy (0), early loss of autonomy (1–2), and significant dependence (≥ 3). Psychometric validation supports a good overall fit (χ²/df = 3.31, CFI = 0.98, TLI = 0.96, RMSEA = 0.09, SRMR = 0.06) [41].

### Phases of the study

#### Translation and adaptation

After obtaining permission to reproduce and validate the scale from the CeASAR at Boston Children’s Hospital [39], the CRAFFT 2.1 underwent a reformulation process, starting with a translation from English to Moroccan dialect, which was subsequently reviewed and revised by a panel of experts including the first two authors, two psychiatrists, two psychologists, and one epidemiologist. To ensure accuracy, the revised version was back translated into English by two independent translators who were unfamiliar with the CRAFFT scale. English experts reviewed the back-translation, provided feedback, and made necessary corrections. Once the revisions were satisfactory, the committee finalized the Arabic dialect version of the scale.

In light of the local context, some experts raised concerns about the translation of the first question: “Have you ever ridden in a CAR driven by someone (including yourself) who was ‘high’ or had been using alcohol or drugs?” They recommended including other common modes of transportation, such as motorcycles, frequently used by young people. Consequently, the question was modified to: “Have you ever ridden in a car or motorcycle driven by someone (including yourself) who was high, had been using alcohol or drugs?“. Additionally, to enhance cultural adaptation and clarity, the phrase “using alcohol or drugs” was replaced with “while drunk or high” ensuring the question remains linguistically and contextually appropriate for the target population.

Finally, the measure underwent pilot testing with 15 participants who used alcohol and drugs. During this test, participants completed the questionnaire and provided feedback, which confirmed that the scale was clear and not misleading, with no issues reported. As a result, no additional revisions were needed after the pilot test.

#### Confirmatory validation

Given the consistently confirmed unidimensional factor structure of the CRAFFT scale across all validation studies, regardless of language or adaptation context [21, 23, 25, 27, 37, 38, 42–46], an Exploratory Factor Analysis (EFA) was not performed [47, 48]. Instead, a CFA was directly conducted to evaluate the fit of the theoretical model to the data within the Moroccan context.

The confirmatory study, conducted from February 2021 to June 2022, included 302 adolescents and young adults with substance use disorders, recruited from a substance use treatment center. This phase aimed to assess the accuracy and reliability of the CRAFFT tool in identifying substance use behaviors among a broader vulnerable youth population, further confirming its well-established unidimensional structure.

## Data analysis

Statistical analyses were conducted using JASP software (version 0.17). Descriptive statistics were employed to summarize participant characteristics. The suitability of the correlation matrix for factor analysis was assessed using the Kaiser-Meyer-Olkin (KMO) test and Bartlett’s test of sphericity [49]. A KMO value above 0.60 confirmed sample adequacy [48, 50, 51], and a significant Bartlett’s test indicated sufficient inter-item correlations for factor analysis [52].

### CFA

CFA was performed using the Weighted Least Squares Mean and Variance adjusted (WLSMV) estimator, chosen for its effectiveness with binary data [47]. The WLSMV estimator is specifically designed for categorical variables, providing accurate parameter estimates by accounting for the non-continuous nature of binary data [53, 54]. It does not assume normality, making it robust to distributional deviations [55], and adjusts for the constrained variance of binary variables, ensuring reliable factor loading estimates [54]. The communality of the objects has been set at 0.40; items with a communality less than this threshold will be removed.

### Model fit assessment

The model’s fit was assessed using multiple indices, including the Comparative Fit Index (CFI), Tucker-Lewis Index (TLI), Normed Fit Index (NFI), and Goodness-of-Fit Index (GFI), where values ≥ 0.90 indicate an adequate fit [34, 56, 57]. Additionally, the chi-square to degrees of freedom ratio (χ²/df) was evaluated, with values ≤ 3 deemed acceptable [58].

Model-data consistency was further examined through the Standardized Root Mean Square Residual (SRMR), where values ≤ 0.05 signified minimal discrepancy [58]. Lastly, the Root Mean Square Error of Approximation (RMSEA), with values ≤ 0.08, confirmed the model’s alignment with empirical data [59–61].

### Reliability and validity

The internal reliability and consistency of the concept were assessed using the KR-20 coefficient for dichotomous items. The overall KR-20 and item-specific values were calculated, with KR-20 values ≥ 0.80 indicating excellent internal consistency [62].

To assess the convergent and discriminatory validity, CRAFFT scores were compared to two gold standards: the Mini International Neuropsychiatric Interview (MINI), specifically the section on alcohol (dependence/use) [40] and the HONC (Hooked on Nicotine Checklist) scale [63].

### Criterion validity and ROC analysis

The CRAFFT’s receiver operating characteristic (ROC) curve was utilized to identify the appropriate cutoff scores in contrast to the MINI scale while evaluating criteria validity. Sensitivity and specificity were the major metrics utilized to select these cutoff values, with Youden’s J index (J = sensitivity + specificity − 1) used to balance the two measurements. The Area Under the Curve (AUC) was employed to evaluate the CRAFFT’s capacity to differentiate between individuals with and without a diagnosis, with a higher AUC value (closer to 1) indicating enhanced discriminative ability [54, 64].

## Ethical aspects

Prior to participating in the research, each participant provided informed consent. The Ethics Committee of Hassan II University Hospital in Fez reviewed and approved the study protocol, including its methods and ethical considerations. Additionally, the study received received authorization from the regional health and social protection directorate for data collection at a substance use treatment center in Fez. This authorization was obtained to ensure full compliance with local legislation and regulatory requirements.

## Results

A total of 380 questionnaires were distributed. After filtering the data and excluding invalid or unreliable responses, 302 valid responses were retained.

The sample was predominantly male (86.42%, *n* = 261), with females representing 13.58% (*n* = 41), resulting in a male-to-female ratio of 6.36:1. The mean age of participants was 18.02 ± 2.34 years, with the following age distribution: 12–13 years (4.63%, *n* = 14), 14–16 years (19.54%, *n* = 59), 17–19 years (43.71%, *n* = 132), and 20–21 years (32.12%, *n* = 97). In terms of educational attainment, the majority had completed secondary education (57.95%, *n* = 175), followed by higher education (22.85%, *n* = 69), primary education (16.88%, *n* = 51), and a small proportion were illiterate (2.32%, *n* = 7). Regarding residential distribution, the majority resided in urban areas (81.46%, *n* = 246), while 6.95% (*n* = 21) lived in rural areas and 11.59% (*n* = 35) in suburban areas. (Table 1).

**Table 1:** Demographic and biographical information

Variable	Category	*CFA *(*n* = *320)*	
		Frequency	%
**Sex**	Female	41	13.58
	Male	261	86.42
Age*	12–13	14	4.63
	14–16 17–19 20–21	59 132 97	19.54 43.71 32.12
**Level of education**	Illiterate Primary education Secondary education Higher Education	7 51 175 69	2.32 16.88 57.95 22.85
**Living environment**	Rural Urban suburban (village)	21 246 35	6.95 81.46 11.59

* The average ages were **18.02** ± **2.34**†

† (Mean ± SD)

Table 2 details the percentages of positive responses provided by participants to the items on the CRAFFT scale, alongside the rates of excessive alcohol consumption among participants who achieved the maximum CRAFFT score. The results indicate a consistent pattern in the percentage of affirmative responses across gender and age groups for specific CRAFFT items. The “Forget” item had the highest proportion of affirmative responses, with an average of 84.93%, followed by the “Alone” item, which received 82.86% positive replies on average.

**Table 2:** Proportion of positive responses on individual CRAFFT-items and excessive alcohol use

	CRAFFT Items / %Yes						Excessive alcohol use (%) *
	Car	*Relax*	*Alone*	Forget	Friends	Trouble	
Total sample (N = 302)	65.23	63.58	81.12	83.44	64.9	58.28	36.75
Males (N = 261)	65.13	61.3	79.69	83.14	63.98	56.7	48.78
Females (N = 41)	65.85	78.05	90.24	84.36	70.73	68.29	34.86
Age*							
12–13 (n = 12)	66.67	66.67	83.33	91.67	58.33	50	25
14–16 (n = 55)	69.09	69.1	85.45	87.27	74.54	72.72	40
17–19 (n = 111)	61.26	59.46	81.98	85.58	64.86	58.56	36.94
20–21 (n = 124)	66.94	64.51	78.23	79.03	61.29	52.42	36.29

* Participants with a high score (6 points)

The “Relax,” “Car,” and “Friends” items showed comparable proportions of “yes” responses, with averages of 66.10%, 65.73%, and 65.52%, respectively. In contrast, the “Trouble” item registered a lower average, with 59.57% affirmative responses across all categories.

36.75% of the study population exhibits excessive alcohol consumption, with 48.78% among males and 34.86% among females. The age group of 14–16 years shows the highest rate of problematic drinking, with 40% compared to other age groups.

Individuals in our sample began using alcohol at an average age of 15.23 years, with a standard deviation of 2.11 years. The substantial standard deviations suggested significant variability in participants’ daily substance use. On average, alcohol was consumed on 81.51 ± 78.99 days, cannabis and its derivatives on 153.91 ± 78.99 days, and other substances (such as pills and prescriptions) on 24.53 ± 72.69 days, as shown in Table 3.

**Table 3:** Initiation age and daily substance use frequency

	Initiation age	Alcohol	Cannabis and derivatives	Other substances
N	302	302	302	302
Average	15.23	81.51	153.91	24.53
Standard deviation	2.11	78.99	149.31	72.69
Minimum	12.00	10.00	0.00	0.00
Maximum	21.00	333.00	365.00	365.00

### Confirmatory factor analysis results

Prior to conducting the factor analysis, the KMO test and Bartlett’s test of sphericity were used to evaluate sampling adequacy and factorability. The overall KMO value was 0.83, with individual component KMO values ranging from 0.76 to 0.89, well above the acceptable threshold of 0.60. Bartlett’s test confirmed sufficient inter-item correlations for factor analysis (χ² = 530.07, df = 15, *p* < 0.001).

Using the WLSMV estimator and a factor loading threshold of 0.40, a unidimensional factor model was selected for the Moroccan version of the CRAFFT 2.1 scale. This model confirms the theoretical structure previously identified in prior studies, with an eigenvalue greater than 1 (= 3.06). (Table 4).

**Table 4:** Factor structure of the Moroccan version of CRAFFT (6 items)

Items	Factor		h^2^	Item-total correlation	KR-20
	Loadings	MSA^*^			
Car	**0.69**	0.82	0.48	0.60	0.76
Relax	**0.53**	0.89	0.28	0.48	0.79
Alone	**0.58**	0.88	0.34	0.53	0.78
Forget	**0.54**	0.84	0.30	0.48	0.79
Friends	**0.87**	0.76	0.76	0.75	0.73
Trouble	**0.62**	0.88	0.38	0.55	0.78
Average	-	**0.83**	-	-	**0.80**

^*^MSA: Measure of Sampling Adequacy

#### Reliability

The KR-20 coefficient for dichotomous items was used to assess internal reliability and consistency. For each item, the KR-20 and inter-item correlations were calculated, as detailed in Table 4. The overall KR-20 coefficient was 0.80, with individual item values ranging from 0.73 to 0.78, demonstrating good internal consistency. Strong inter-item correlations further support the reliability of the Moroccan CRAFFT scale.

#### Convergent validity

**Fig. 1:**
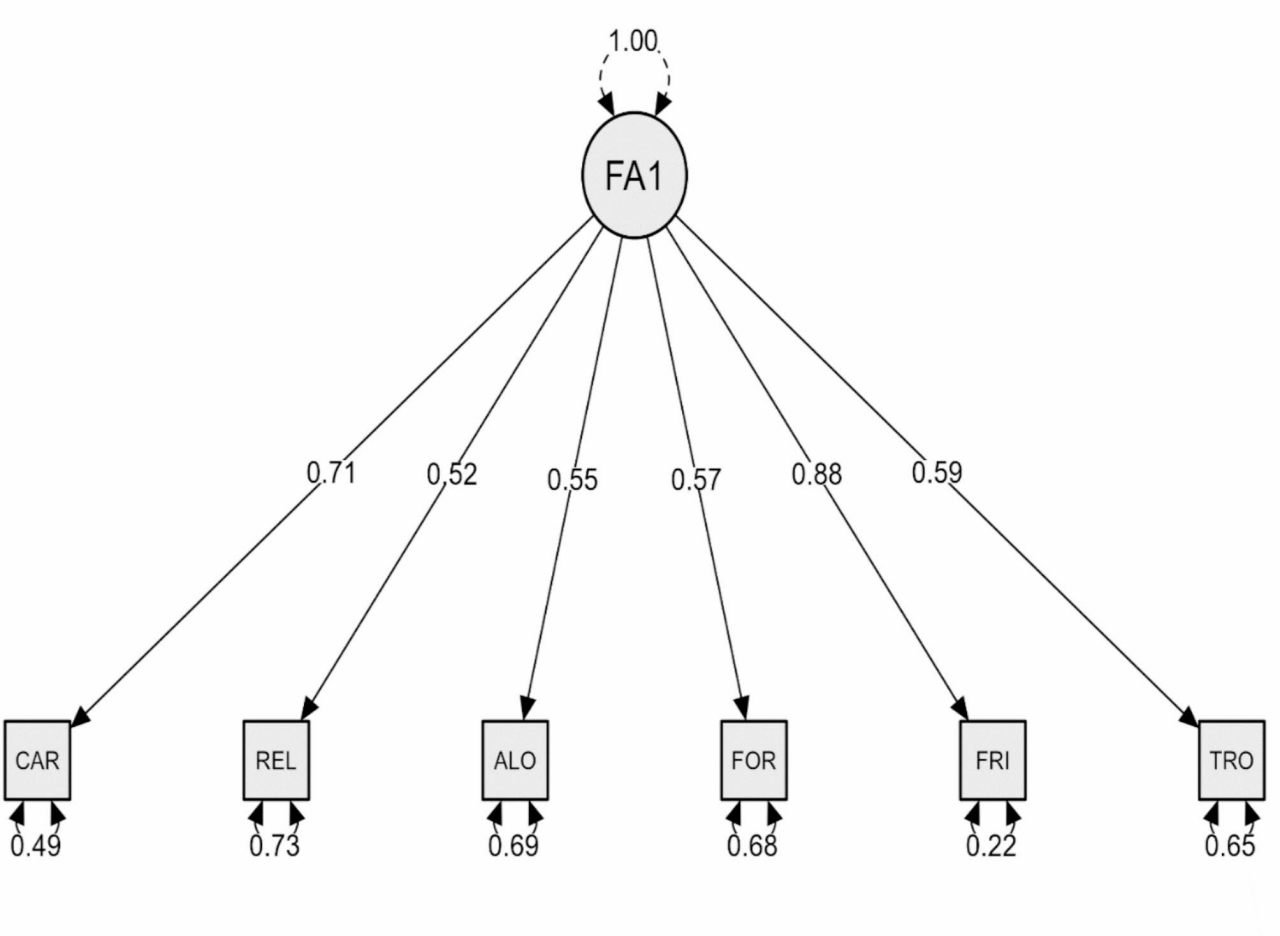
CFA measurement model **FA** Factor; **CAR** Car item; **REL** Relax; **ALO** Alone; **FOR** Forget; **FRI** Freinds; **TRO** Trouble

The CFA results revealed that the standardized regression coefficients were consistently above 0.52. Notably, the “friends” component had the greatest factor loading, with a value of 0.88. These results, with regression coefficients over the 0.50 criterion, support the convergent validity of the first-order CFA. (Fig. 1).

Additionally, correlation analysis between the CRAFFT instrument and the MINI Gold Standard assessment showed a strong and statistically significant association (*r* = 0.82, *p* < 0.001), as shown in Table 5. This strong correlation signifies a high degree of alignment between the two measures, validating the effectiveness of the CRAFFT and supporting its convergent validity.

**Table 5:** Correlation between CRAFFT, MINI, and HONC scores

	CRAFFT			
	Pearson’s *r*	*p*	Lower 95% CI	Upper 95% CI
**MINI**	0.81***	< 0.001	0.76	0.84
**HONC**	0.20	< 0.001	0.09	0.30

*p < 0.05, ** *p* < 0.01, *** *p* < 0.001

#### Discriminant validity

By comparing the CRAFFT scale with the HONC scale, which specifically assesses nicotine dependence and loss of autonomy linked to smoking, the discriminant validity of the CRAFFT scale was assessed. The Pearson correlation coefficient (r) showed that the overall scores of the CRAFFT scale and the HONC scale were lowly correlated (*r* = 0.20, *p* < 0.001), indicating that the two scales measure distinct concepts (Table 5*).* This result confirms the discriminant validity of the CRAFFT, demonstrating that it offers a broader assessment of substance use risk rather than measuring nicotine dependence alone.

#### Fitness of the one-factor structure

The CFA results demonstrate the robustness of the unidimensional factor model (Table 6). Satisfactory fit is indicated by the chi-square to degrees of freedom ratio (χ2/df) of 1.91, a CFI of 0.98 (> 0.90), and a GFI of 0.98 (> 0,90). The absence of significant discrepancy between the observed data and the model is supported by a SRMR of 0.03 (< 0.05) and a RMSEA of 0.06 (< 0.08). The TLI, NNFI, and NFI indices, all at 0.97 (> 0.90), confirm the model’s adequacy.

**Table 6:** Fit indices of the one-factor structure

Fit index	χ^2^/df	CFI	GFI	RMSEA	SRMR	NFI	TLI
*Observed Value*	1,91	0.98	0.98	0.06	0.03	0.97	0.97
*Level of acceptance*	< 3	> 0.90	> 0.90	< 0.08	< 0.05	> 0.90	> 0.90

**χ2** Chi-squared test; **df** Degrees of Freedom; **CFI** Comparative fit index; **GFI** goodness of fit index; **RMSEA** root mean square error of approximation; **SRMR** Standardized Root Mean Square Residual; **NFI** normed fit index; **TLI** Tucker-Lewis Index

### Detection accuracy

The assessment of CRAFFT detection capabilities using the MINI as the Gold Standard shows strong sensitivity (78.35%) and a Positive Predictive Value (PPV) exceeding 0.90. The greatest Youden index (Y = 0.70) indicates that a cutoff of 4 provides the best balance of sensitivity and specificity for the CRAFFT scale. (Table 7).

**Table 7:** CRAFFT screening metrics across multiple cutoff points

Cut-off	Se (%)	Sp (%)	PPV (%)	NPV (%)	Y	AUC	METRIC
1	100	0	84.11	-	0.00	0.89	1.00
2	95.67	31.25	88.04	57.69	0.27	0.89	1.27
3	85.83	68.75	93.56	47.83	0.55	0.89	1.55
4^a^	78.35	91.67	98.03	44.44	0.70	0.89	1.70
5	66.14	97.92	99.41	35.34	0.64	0.89	1.64
6	35.04	100	100	22.54	0.35	0.89	1.35

**Se**: sensitivity, **Sp**: specificity, **PPV**: positive predictive value, **NPV**: negative predictive value, and **Y**: Youden index; **AUC**: Area Under the Curve

**Fig. 2:**
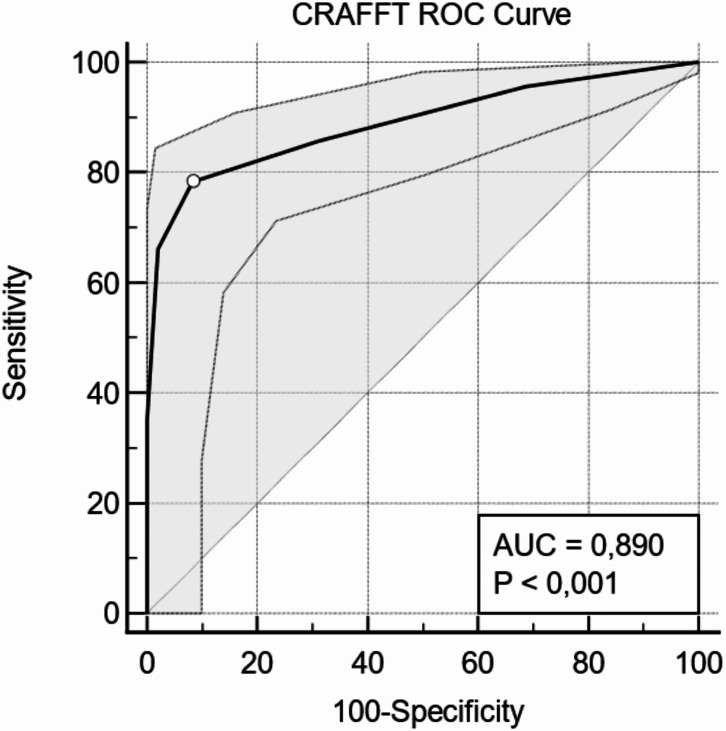
ROC curve and AUC for CRAFFT

The ROC (Receiver Operating Characteristic) curve analysis confirms the high discriminatory power of the assessment scale. As illustrated in Fig. 2, the AUC (Area Under the Curve) value was found to be 0.89, with a 95% confidence interval ranging from 0.83 to 0.92. This AUC finding confirms the instrument’s strong ability to differentiate between individuals with a clinical diagnosis and those without.

## Discussion

This study aimed to assess the psychometric properties and validate the CRAFFT2.1 scale, a Moroccan adaptation designed to identify alcohol and drug use that could lead to negative health or social consequences. A 302 individuals with alcohol and drug use disorders were included, recruited from a substance use treatment center in Fez.

The study sample was predominantly masculine, with 86.42% males and 13.58% females, and a mean age of 18.02 years (SD = 2.34). The average age at which participants first began drinking alcohol was 15.23 years (SD = 2.11). These characteristics confirm established findings, particularly the higher prevalence of substance use among males compared to females, both in Morocco [9, 12, 65] and globally [5, 66, 67], as well as the early initiation of substance use, generally around the age of 15, which is consistent with established patterns observed in similar studies [5].

The specific context of the study—conducted within a substance use treatment center —likely accounts for the considerable variation in daily substance use observed among participants, with high averages reported for alcohol (81.51 ± 78.99 days), cannabis and its derivatives (153.91 ± 78.99 days), and other substances (24.53 ± 72.69 days). In contrast to studies involving general populations, where variability in substance use is typically more predictable [21, 46], our participants, due to their clinical conditions, exhibit more intensive and diverse patterns of substance use.

The “Forget” item showed the highest affirmative response rate (84.93%), followed by “Alone” (82.86%). The “Relax” (66.10%), “Car” (65.73%), and “Friends” (65.52%) items had similar rates, while “Trouble” had a lower rate (59.57%), underscoring the scale’s efficacy in detecting high-risk youth and clinical sensitivity [21, 46]. However, our findings surpass those of earlier studies using the same tool [27, 43, 46], likely due to our sample’s composition: individuals in treatment for substance use disorders, indicating higher frequency and severity of use. In contrast, prior studies involved more diverse samples of adolescents and young adults, including occasional users.

The internal structure of the Moroccan version of the CRAFFT was confirmed through a unidimensional factor model, demonstrating robust internal reliability and consistency, in alignment with prior research [21, 23–25, 27, 28, 37, 38, 44–46]. The KR-20 coefficient for our model was 0.80, surpassing the validated values of 0.55 for the German version [28], 0.68 for the Spanish version [24], and 0.73 for the Asian version [27], though it was lower than the 0.85 reported for both the Nigerian [44] and Korean versions [45]. Furthermore, the standardized factor loadings for the Moroccan model, which ranged from 0.53 to 0.88, were comparable to those observed in the Asian version (0.60 to 0.93) [27] and exceeded the loadings reported for the German (0.36 to 0.69) [28] and Spanish/Argentinian (0.47 to 0.85) versions [24] of the CRAFFT.

The convergent validity of our Moroccan version of the CRAFFT was strongly supported by its high and statistically significant correlation with the MINI gold standard (*r* = 0.82, *p* < 0.001), indicating a substantial alignment between the two measures [21, 27, 37, 42]. Conversely, the low correlation of the CRAFFT with the HONC gold standard (*r* = 0.20, *p* < 0.001) underscores its ability to measure different constructs, thus confirming its discriminant validity and its broader applicability in evaluating substance use risk beyond nicotine dependence [24, 26].

Our one-factor model demonstrated robust fit indices, with a satisfactory χ2/df ratio of 1.91, CFI of 0.98, GFI of 0.98, SRMR of 0.03, RMSEA of 0.06, and TLI, NNFI, and NFI all at 0.97, demonstrating the model’s adequacy and consistency with results from other CRAFFT versions [24, 26, 28, 46].

A cutoff score of 4 or higher on the CRAFFT proved optimal for our population, providing a strong balance between sensitivity and specificity, with a Youden index of 0.70. This higher cutoff also enhances the CRAFFT’s detection performance, with sensitivity and Positive Predictive Value both exceeding 0.90. These results contrast with previous research recommending a threshold of 2 to 3 for identifying at-risk adolescents [21, 23, 26, 37, 39, 43, 68]. However, studies conducted in France [42] and Argentina [24] also identified a cutoff score of 4 as optimal. These variations may reflect differences in cultural contexts or patterns of substance use across populations, which can influence the appropriateness of different thresholds for risk assessment.

Several limitations of this study must be considered when interpreting the results. Firstly, the specific context of the studied population—adolescents and young adults undergoing treatment at a substance use treatment center —limits the generalizability of the results to the broader Moroccan adolescent and young adults population. The characteristics and needs of this clinical group may differ significantly from those of community samples, affecting the applicability of the findings. Secondly, the relatively small sample size of the study may have influenced the determination of the optimal detection threshold. To validate these results, it is crucial to replicate the study with larger and more diverse samples of Moroccan adolescents and young adults. Moreover, the exclusive reliance on self-reported data introduces potential biases, such as social desirability or missing information. The validity of the conclusions could be enhanced by incorporating additional data sources, such as clinical interviews or administrative records. Furthermore, although the CRAFFT scale is widely used elsewhere, its applicability in the Moroccan context remains limited due to the absence of comparative validation with a recent and adapted tool, such as the eleven-item Alcohol, Smoking and Substance Involvement Screening Test (ASSIST-11) [69]. This brief tool, validated in 42 countries, assesses a broader range of substances and demonstrates strong psychometric properties [70]. This limitation highlights the need for future research to explore the complementary use or comparative validity of these tools in Morocco across diverse cultural and clinical settings. Finally, the cross-sectional design of the study restricts the assessment of the CRAFFT’s predictive validity in identifying adolescents and young adults at risk of developing long-term substance use problems. Longitudinal studies are needed to explore this aspect further and to better understand the stability and effectiveness of the optimal threshold over time.

## Conclusion

This study confirms the psychometric robustness of the Moroccan version of the CRAFFT 2.1 scale, demonstrating its validity and reliability for screening alcohol and substance use behaviors among adolescents and young adults. Despite certain limitations, the findings highlight its strong screening capabilities, suggesting that CRAFFT 2.1 is effective not only in clinical settings but also for large-scale implementation in the general population. These results underscore its potential for informing prevention strategies and public health policies.

## Electronic Supplementary Material

Below is the link to the electronic supplementary material.

Supplementary Material 1 ( Database)

## Data Availability

No datasets were generated or analysed during the current study.

## Abbreviations

AUC: Area Under the Curve
AGFI: Adjusted Goodness-of-Fit Index
ASSIST: Alcohol, Smoking and Substance Involvement Screening Test
CFA: Confirmatory Factor Analysis
CeASAR: Center for Adolescent Substance Abuse Research
CFI: Comparative Fit Index
CRAFFT: Car, Relax, Alone, Forget, Friends, Trouble
EFA: Exploratory Factor Analysis
GFI: Goodness of Fit Index
GSHS: Global School-Based Student Health Survey
HONC: Hooked on Nicotine Checklist
KMO: Kaiser-Meyer-Olkin
KR-20: Kuder-Richardson Formula 20
LMICs: Low- and Middle-Income Countries
MedSPAD III: Mediterranean School Project on Alcohol and Other Drugs
MINI: Mini International Neuropsychiatric Interview
NFI: Normed Fit Index
NNFI: Non-Normed Fit Index
NPV: Negative Predictive Value
PAS: Psychoactive Substances
PPV: Positive Predictive Value
ROC: Receiver Operating Characteristic
RMSEA: Root Mean Square Error of Approximation
Se: Sensitivity
Sp: Specificity
SRMR: Standardized Root Mean Square Residual
TLI: Tucker-Lewis Index
UNODC: United Nations Office on Drugs and Crime
WLSM: Robust Weighted Least-Squares Estimator
Y: Youden Index
